# Complexity of crystalline lens wobbling investigated by means of combined mechanical and optical simulations

**DOI:** 10.1364/BOE.488176

**Published:** 2023-05-02

**Authors:** Agnieszka Boszczyk, Fabian Dębowy, Agnieszka Jóźwik, Ali Dahaghin, Damian Siedlecki

**Affiliations:** 1 Department of Optics and Photonics, Wroclaw University of Science and Technology, Wybrzeze Wyspianskiego 27, 50-340 Wroclaw, Poland; 2 Currently with the Medical Technology Research Centre, Faculty of Health, Education, Medicine and Social Care, Chelmsford Campus, Bishops Hall Lane, Chelmsford CM1 1SQ, UK

## Abstract

Crystalline lens wobbling is a phenomenon when the lens oscillates briefly from its normal position immediately after stopping the rotational movement of the eye globe. It can be observed by means of Purkinje imaging. The aim of this research is to present the data and computation workflow that involve both biomechanical and optical simulations that can mimic this effect, aimed to better understanding of lens wobbling. The methodology described in the study allows to visualize both the dynamic changes of the lens conformation within the eye and its optical effect in terms of Purkinje performance.

## 1. Introduction

The eye undergoes rapid rotational movement as a result of changing the gaze. When the eye stops, immediately after this rapid motion, the crystalline lens manifests an inertial movement. This movement, also referred to as lens wobbling, is likely to be a superposition of three basic movements induced by the rapid eye’s rotation: an oscillatory angular tilt of the lens, an axial displacement and a lateral dislocation that is tangential to the angular direction of the eye’s rotation. The latest scientific reports describe the possibility of observation of the effect of the dynamical behaviour of the crystalline lens induced by rapid changes of gaze direction in the form of the movement of Purkinje's reflection from the back surface of the crystalline lens [1–3].

In general, Purkinje images are reflections of light from several optical interfaces of the optical system of the eye. The first two Purkinje images come from the anterior and the posterior corneal surfaces (PI and PII, respectively) and the others are results of reflection from the front (PIII) and back (PIV) surfaces of crystalline lens, respectively.

**Fig. 1.**
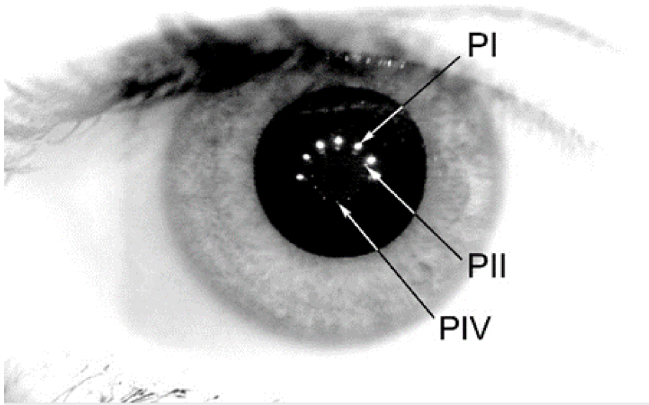
An illustrative example of Purkinje images captured in the healthy human eye in vivo. Images PI, PII are the reflections from anterior and posterior cornea, respectively. Image PIV, associated with the posterior crystalline lens, is inverted in relation to PI and PII. Image PIII is indistinguishable. Contrast of the image was enhanced for better visibility of PIV.

In healthy emmetropic eyes, PI, PII and PIV are almost specular reflections (Fig. 1), whilst PIII appears diffused and speckled in its structure [4]. Also, in terms of Gaussian image planes, for healthy eyes PI, PII and PIV are formed at roughly the same distance from the anterior pole of the cornea (3.6 mm, 3.9 mm and 4.6 mm, respectively), while the axial distance of PIII alters significantly (10.6 mm) [5]. This difference in axial locations results in the fact that, while the axial positions of PI and PIV usually meet the depth of focus range of a typical telecentric lens used for observations, the axial position of PIII usually outreaches this range. Moreover, the size of PIII is relatively large. Due to its high magnification, while changing the lens position (its tilt and/or lateral displacement), PIII rapidly goes beyond the area of interest (usually associated with the pupil) and is markedly truncated by the iris. All these features make the PIII images barely visible in normal, healthy eyes, if at all, and it is particularly difficult to register and to process for further investigations [6]. For all these reasons, the wobbling of the crystalline lens is commonly observed only in the form of PIV movement, and not the mutual movements of PIII and PIV.

The literature review [7,8], as well as our experience show that both the PIII and PIV images in the eyes with the intraocular lens (IOL) are bright and clear enough to be captured at once. This effect can be explained on the basis of differences in the geometry and material properties of the natural lens and the IOLs. Therefore, those reflections find widespread use in unambiguous determination of IOL position after cataract surgery, namely: estimation of its axial and lateral positions and tilt [7–11]. Some authors use them to measure intraocular scattering as well, to provide information on the total scattered light from different ocular structures [6,12].

In studies dealing with the observation of lens wobbling by means of PIV image movement, it is usually assumed that the lens can move transversely to the optical axis, but its inclination is constant [1]. In such a situation, knowing the optical parameters (radii of curvature, thicknesses, refractive indices) makes it possible to determine the lens displacement just based on the relative PIV and PI image positions.

The phenomenon of wobbling is still not fully understood and seems to be much more complex in nature. In the current work, we hypothesize that the wobbling of the crystalline lens consists of both movements: its lateral displacement (decentration) and tilt. Although the coexistence of these two basic motions in the dynamic wobbling effect may seem obvious and even trivial, except for general suggestions presented by He et al. [3] and Tabernero et al. [13], it has never been studied in details either experimentally or numerically.

The main goals of the study was to numerically prove the coexistence of tilt and decentration in the lens wobbling motion and to explore the complex nature of this phenomenon. For this purpose we have developed the data and computation workflow, based on both the mechanical simulations of the wobbling inertial motion dynamics (by means of finite elements modeling, FEM) and optical simulations of Purkinje performance, where the actual location and inclination of the crystalline lens within the eye globe were considered. This approach is based on strong physical grounds of Purkinje imaging technique as the optical projection being the result of the actual spatial conformation of the crystalline lens with regard to the optical axis of the eye. In other words, Purkinje performance is a result of superposition of spatial localization parameters: tilt and decentration of the crystalline lens. Both of these parameters are subject to change as a result of inertial motion induced by eye globe rotation, and both contribute to the effective Purkinje performance. The assumption of the analysis was that the simulations should reflect the capabilities of the actual measurement system (and the aforementioned limitations related to the possibility of recording the PIII image, as well). Thus, the considerations were limited to the observation of only the first and the fourth Purkinje reflections (PI and PIV, respectively). Going further, we also demonstrate that the presented computation workflow is responsive to the magnitude of biomechanical parameters, characterizing a given structure (i.e. Young’s modulus of zonular fibres) and the magnitude and variability over time of the Purkinje performance output may be affected by these parameters.

## 2. Materials and methods

### 2.1 Geometry and material properties of the model eye

**Fig. 2.**
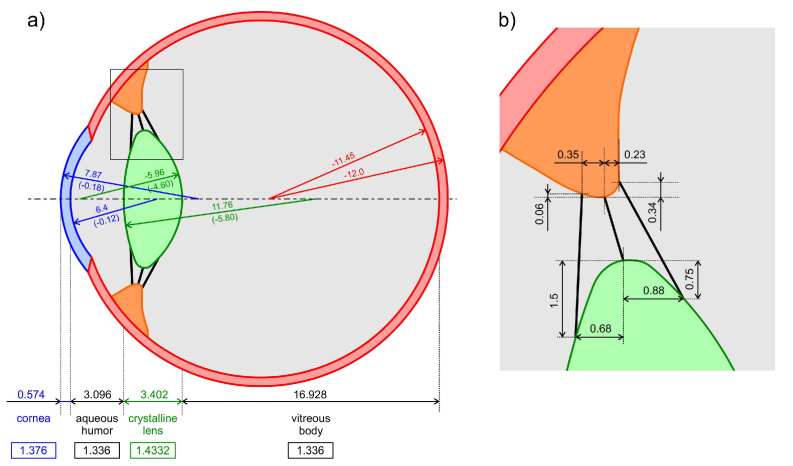
a) Selected geometrical and optical parameters of the human eye model used for the purpose of simulations in the current study. The optical media are noted in the lower part of the figure. Additionally, ciliary body is the area marked with orange, and zonular fibers are the black lines joining the ciliary body with the crystalline lens. Distances between the surfaces and their radii of curvature are given in millimeters. Respective conic constants are given in brackets. The numbers in rectangles represent the respective refractive index of the media. The zonular region marked with rectangle is magnified in part b) of the figure and present details of the arrangement of the ligaments attachments to the crystalline lens and ciliary body.

In order to assess the influence of material properties of the zonules on the lens wobbling amplitude and the optical performance of this wobbling in terms of Purkinje imaging, both biomechanical and optical simulations were performed. The methodology of simulations and sequence of processing the data will be presented in detail in the next paragraph. The starting point for both these types of simulations was the assumption regarding the geometry of the model eye used for their purpose. We decided to implement the geometry of the human eye model introduced by Zapata-Díaz et al. [14]. It was developed on the basis of experimental biometric data, mainly for the purpose of optical simulations and analysis. Moreover, its age and accommodation dependency makes it suitable to add up to geometrical basis for other applications, including mechanical simulations. For the purpose of the current study we implemented the geometry of the 20 year old unaccommodated eye. Both geometrical and optical parameters describing the several elements of the model are presented in Fig. 2.

Some additional data were necessary to adapt to the model for the purpose of dynamic mechanical simulations. The two-dimensional FEM model of the human eye contains the main and the most influential components in eye biomechanics: lens, zonular fibers, cornea, sclera, and ciliary body that undergoes 15 mmHg intraocular pressure as an incompressible, viscous and Newtonian fluid. In the physiological human eye, two thin layers exist: choroid and retina, but their role and contribution to the crystalline lens wobbling appearance seems to be negligible. Therefore, for the purpose of simplification, these layers have been ignored in this study. The lens was modelled to be suspended on three pairs of zonular ligaments: anterior, equatorial, and posterior one, with each ligament having a thickness of 50 µm. The attachment location of the anterior zonule was 0.68 mm anteriorly to the lens equator, and the posterior zonule was 0.88 mm posteriorly. A similar arrangement can be found in [15] and in the work of Lanchares et al. [16], but the exact dimensions may differ due to differences of the overall geometry of the lens. The details of the ligament attachments distribution are presented in Fig. 2(b).

Almost all constituents of the model, but vitreous body and aqueous humour, were assumed to be linearly elastic, isotropic and homogeneous, with different material properties reported in the previous studies for a normal human eye. Both the vitreous body and the aqueous humour were modeled as viscoelastic fluids. Table 1 presents the values of mechanical constants of different structures of the eye that can be found in the literature [17–25]. In case of zonules, a significant spread between the data can be noticed [21,22,26,27]. We adapted the 2 extreme values in order to test the respondability of the simulation and analysis approach to the biomechanical variability of ocular structures.

**Table 1.** Material properties of various structures of the human eye [17–25] (“N.A.” stands for “not applicable”)

Modelled structure	Young’s modulus [MPa]	Poisson’s ratio [-]	Dynamic viscosity [Pa·s]	Density [kg/m^3^]
Sclera [17]	3	0.47	N.A.	1400
Cornea [17]	0.4	0.42	N.A.	1400
Ciliary Muscle [18]	0.82	0.4	N.A.	1600
Lens [19]	1.45	0.47	N.A.	1225
Zonular Fibers	0.279 [21]	0.4	N.A.	1000 [22]
	2.539 [23]	0.4		1000 [22]
Aqueous humor [24,25]	N.A.	N.A.	0.00074	996
Vitreous body [24,25]	N.A.	N.A.	0.00074	1000

### 2.2 Methodology of simulations

#### 2.2.1 Purkinje performance as a function of lens tilt and decentration

At this stage of the study, the optical model of the human eye in the Purkinje imaging system was developed. It was designed to simulate the performance of Purkinje images for different configurations of the crystalline lens alignment within the eye. Numerical simulations of Purkinje images were performed using Zemax OpticStudio [28], using the non-sequential modelling feature of this software.

**Fig. 3.**
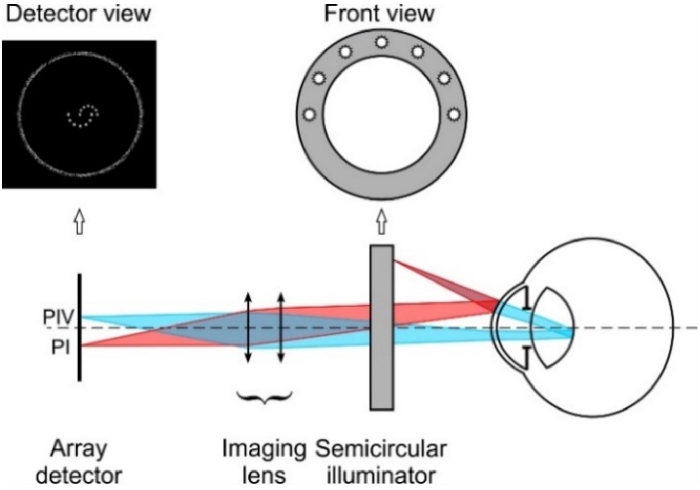
Schematic view of the Purkinje imaging system used for the purpose of simulations.

For the purpose of simulations, the pupil size was assumed to be 8 mm in diameter to make the observation of PIV possible for wide ranges of lens tilt and decentration. As an illuminator, 5-mm diameter diodes in a semicircular configuration were used. The axial position of the illuminator was 260 mm in front of the cornea (Fig. 3).

**Fig. 4.**
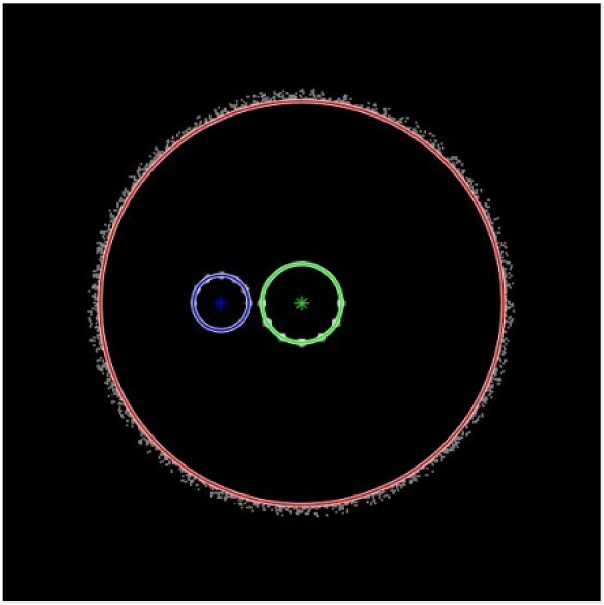
An example of simulated detector view of Purkinje images PI (associated with the green circle) and PIV (associated with the blue circle).

Having the model of the setup implemented in the optical analysis software, the Purkinje images were simulated on bitmaps with single pixel size of 3.6 µm × 3.6 µm, which corresponds to the observed object size 18 µm × 18 µm (as the magnification of the objective lens was assumed to be 0.2). It needs to be emphasized that the Purkinje images positions were calculated as the positions of the center of the circle associated with a PI and PIV images of a semicircular illuminator (Fig. 4) and are scaled to the real size (pixel number × 18 µm).

In this step of study, the relative PI-PIV performance was estimated for lens decentrations and tilts. The following ranges of lens dislocation were considered: 0-1.5 mm (with the step of 0.1 mm) for decentration and -7.5 deg to +7.5 deg (with the step of 0.5 deg) for tilt. These ranges were reasonably selected on the basis of the literature analysis related to the lens wobbling [9].

We decided to neglect the axial displacement of the lens for further considerations. From the optical point of view, this type of motion affects the PIV image size. Our initial simulations showed that changing the position of the lens by 1 mm into the eye causes the changes of the radius of the PIV image by 7.2 µm. It seems barely possible to observe this effect due to crystalline lens inertia by means of Purkinje imaging. Additionally, because of the axial symmetry of the eye model in its initial configuration, the torsional movement was neglected as well, and only the *x*-coordinate and rotation about the *y* axis were changed in simulations. The rotation of the whole eyeball was not taken into account, so the pupil and PI position remained stationary.

#### 2.2.2 FEM simulations of lens wobbling motion

The model of the eyeball presented in details in the previous paragraph (see Fig. 2 for geometry and Table 1 for material properties) was implemented to finite element method (FEM) with use of COMSOL Multiphysics software (version 5.6). In total, mechanical model of the eye consisted of 13234 triangular elements with an average element quality of 0.78 (see Table 2 for details of the elements quality data). This quality measure was estimated using COMSOL Multiphysics’s built-in quality assessment that provides the rating between 0 and 1, and the quality parameter larger than 0.5 was considered fine [19].

**Table 2.** Mesh data of the FEM model

Structure of the eye globe model	Number of elements	Minimum element quality	Average element quality	Element area ratio	Mesh area [mm^2^]
Sclera	656	0.47	0.74	0.104	35.94
Cornea	84	0.52	0.75	0.25	5.43
Ciliary Muscle	588	0.61	0.85	0.007	6.58
Lens	1936	0.60	0.87	1.3E-4	22.06
Zonular Fibers	432	0.18	0.70	0.007	0.56
Aqueous humor	1525	0.58	0.86	0.007	29.82
Vitreous body	8013	0.21	0.87	8.9E-5	355.3

In mechanical simulations, the whole eyeball was rotated azimuthally by 9 degrees around the axis perpendicular to the plane of the drawing in Fig. 2. The distance between this axis of rotation and the corneal apex was assumed to be 12.55 mm into the eye. According to the data provided by Collewijn et al. [29], the angular velocity of the eye during rotation is 5.67 rad/s for 10 deg maximum relative rotation. We implemented this data in simulations of the eye dynamics, so that the whole eye rotation lasted 28 ms. The rotational movement of the whole eye globe evoked the inertial motion of the crystalline lens, which was of particular importance in this study. The data on lens motion was recorded in terms of the time evolution of the magnitudes of crystalline lens tilt and lateral displacement estimated for the point of the apex of the anterior lens surface.

Exactly the same procedure of rotation and lens wobbling simulations was adapted to each of two FEM models, which differed only by the magnitude of Young’s modulus of zonular fibres (see Table 1.). This way it was possible to determine what is the response of the coupled opto-biomechanical modeling and simulation tool to the changes of one of the most basic and purely mechanical parameter.

#### 2.2.3 Optical simulations of the lens wobbling performance

Since the lens inertial motion cannot be observed directly, but rather its optical effect by means of Purkinje imaging, the latter stage of the study was implementation of the time evolutions of both tilt and lateral displacement obtained as results of FEM simulations, to the Purkinje imaging setup developed in the first stage of the study in Zemax OpticStudio (see Fig. 3.) and estimate the Purkinje performance for the lens wobbling data simulated for both FEM models described in the previous paragraph. These optical simulations were aimed to estimate, how the mechanical properties of the zonular fibers may affect the crystalline lens wobbling in terms of Purkinje imaging.

**Fig. 5.**
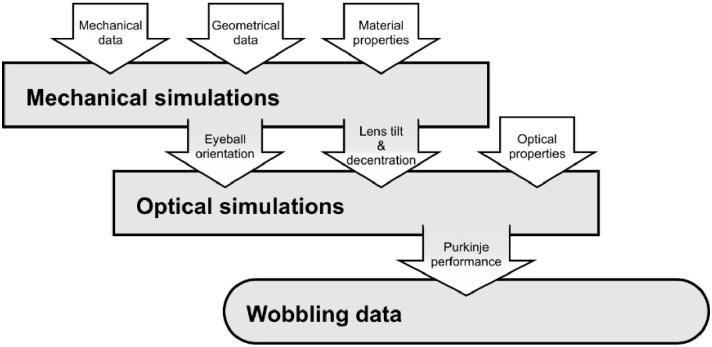
Diagram presenting the general data and computation workflow between mechanical and optical simulations, ultimately leading to the resultant wobbling trajectory data.

The general scheme of the data and computation workflow is presented in Fig. 5.

## 3. Results

### 3.1 Purkinje performance as a function of lens tilt and decentration

In the first step the relative positions of PIV and PI were analysed for different configuration of lens tilt and decentration. The simulations of how decentration and tilt of the lens separately affect the Purkinje images position are presented in Visualization 1 and Visualization 2, respectively.

**Fig. 6.**
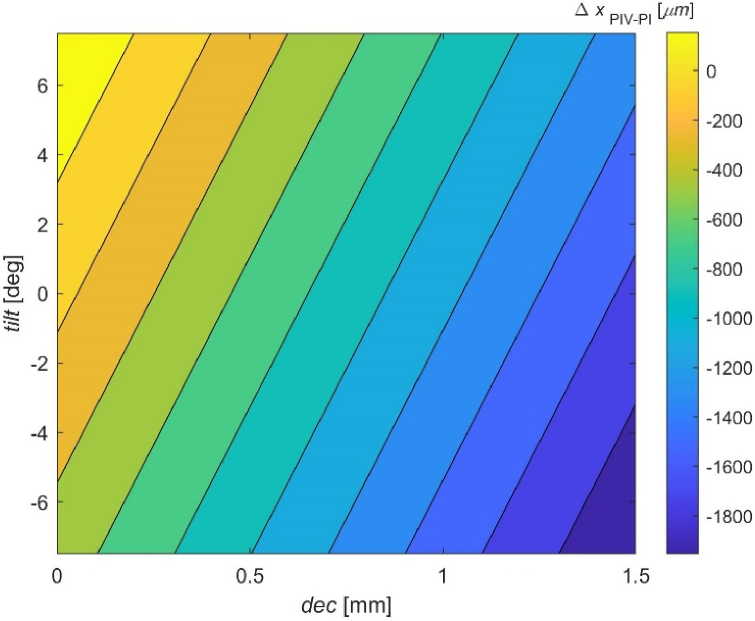
Relative PIV-PI position as a function of crystalline lens decentration and tilt.

The relative position of PIV and PI was plotted against the tilt and decentration of the crystalline lens in Fig. 6. On this contour plot the straight black lines represent the same relative PIV-PI position caused by combination of two components of lens spatial conformation. Since the whole data can be successfully fitted with a plane, these lines are exactly perfectly parallel.

The fitted plane (R^2^ = 0.9999) is given by the following equation:

(1) $$\Delta x_{PIV-PI}=-2.4(\pm 2.0)-1057(\pm 3)\cdot \text{dec}+48.9(\pm 0.3)\cdot \text{tilt},$$

where $\Delta x_{PI-PIV}$ is expressed in micrometers, decentration (*dec*) is expressed in mm, and *tilt* is expressed in degrees. The constant term in Eq. (1) represents the error of numerical simulations. If omitted, the equation can be written in the following form:

(2) $$\Delta x_{PIV-PI}=-1057(\pm 3)\cdot dec+48.9(\pm 0.3)\cdot tilt.$$

It must be noticed that the same position of PIV in relation to PI (contour lines in Fig. 6) can be obtained for many different combinations of lens decentration and tilt. Independently what PIV-PI performance is considered, for every 1 mm of decentration there is 21.62 deg of tilt in the opposite direction required to maintain a stable PIV position in relation to PI (based on Eq. (2)).

In order to estimate the influence of the parameters of the eye on the resulting surface, similar simulations were performed for different values of central corneal thickness, corneally induced refractive error (obtained by change of the anterior corneal curvature) and different distances between the illuminator and the eye. The parameters describing the planes approximating the results of PI-PIV position against decentration and tilt of the lens are listed in Table 3. It can be seen that they are not very different, which means that the parameters of the eye and setup do not change significantly the slopes of the resulting plane.

**Table 3.** Fitted plane equation (PIV-PI position against to decentration and tilt of the lens) for different changes in the model eye

Type of model modification	Fitted plane equation
Basic eye model	$\Delta x_{PIV-PI}=-1057(\pm 3)\cdot dec+48.9(\pm 0.3)\cdot tilt$
Corneal refractive error -2D	$\Delta x_{PIV-PI}=-1056(\pm 3)\cdot dec+49.1(\pm 0.4)\cdot tilt$
Central corneal thickness 0.5 mm	$\Delta x_{PIV-PI}=-1056(\pm 3)\cdot dec+48.8(\pm 0.3)\cdot tilt$
Source distance 250 mm	$\Delta x_{PIV-PI}=-1057(\pm 3)\cdot dec+48.6(\pm 0.3)\cdot tilt$
Anterior chamber depth 3.596 mm	$\Delta x_{PIV-PI}=-1074(\pm 3)\cdot dec+50.2(\pm 0.3)\cdot tilt$

### 3.2. FEM wobbling simulations results

**Fig. 7.**
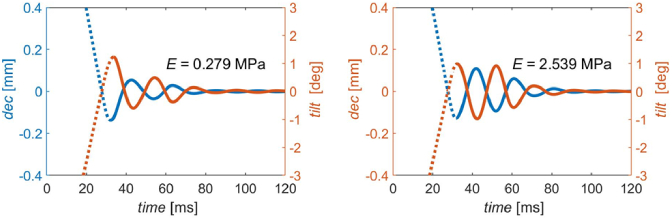
The wobbling effect observed separately as the time evolution of tilt and decentration of the crystalline lens due to horizontal eye movement for eye models with different Young`s modulus of zonular fibres *E*: 0.279 MPa and 2.539 MPa. Both magnitude and time axes were unified in order to ease comparison between the trajectories.

The next step was to determine the behavior of the crystalline lens induced by horizontal eye movement for different mechanical configurations. The plots of tilt and decentration of the crystalline lens as a function of time are presented in Fig. 7. Each curve represents the mechanical simulations performed for different models, depending on the magnitude of the Young’s modulus parameter of the zonular fibers (0.279 MPa and 2.539 MPa). One can notice, that both waveform shapes of tilt and decentration time evolutions are quite similar (almost in counterphase), but not precisely the same. Some amount of phase shift can be observed between the plots for tilt and decentration (see Discussion for details).

### 3.3. Crystalline lens wobbling in terms of Purkinje performance

**Fig. 8.**
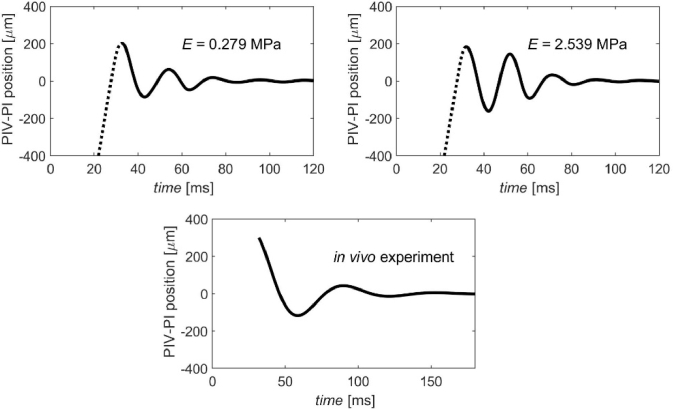
The wobbling effect observed as a change of PIV-PI position for model eye with different Young’s modulus of zonular fibres *E*=: 0.279 MPa and 2.539 MPa. For comparison purposes, the lower panel presents the reconstructed results of *in vivo* measurement of the human eye [12]. Both amplitude and time axes were unified in order ease comparison between the trajectories.

Since our considerations described in subsection 3.1 proved that the relation between the relative PIV-PI distance and both lens tilt and decentration is very straightforward and can be explicitly expressed in terms of analytical equation (Eq. (2)), the wobbling pattern can be estimated with ease. For the waveforms of lens decentration and tilt obtained by means of FEM dynamics simulations, the relative position of PIV and PI image dynamics in time, is presented in Fig. 8.

Table 4 presents the quantitative summary of tilt and decentration trajectories together with Purkinje PIV-PI patterns estimated for both models that differed only by the magnitude of Young’s modulus of the zonular fibres (see Fig. 7 and Fig. 8).

**Table 4.** Peak (*valley – in case of decentrations) appearance times [ms] for the eye model with different values of Young’s modulus of zonular fibres

Peak number	*E *= 0.279 MPa			*E* = 2.539 MPa		
	Tilt	Dec*	PIV-PI	Tilt	Dec*	PIV-PI
1	33.7	32.3	33.0	32.4	31.8	32.1
2	54.3	53.8	54.1	52.0	51.8	51.9
3	74.0	74.1	74.0	71.0	71.1	71.1

For better visualization of the wobbling process, the simulations of lens wobbling motion and the corresponding simulations of Purkinje images is shown in Visualization 3. The simulated lens motion is presented in left panel, while the right panel present the optical effect (Purkinje imaging) of this motion (synchronized). The colormap is associated with absolute displacement of the mesh points within the crystalline lens with respect to the final, balanced position of the lens, when the lens wobbling is stopped.

## 4. Discussion

Due to strong physical background and relative simplicity and compactivity of the optical realizations, Purkinje imaging is still one of the most common imaging techniques used to investigate the dynamics and activity of the oculomotor system. Imaging instruments based on this technique are considered to be reliable tools to provide quantitative data on spatial alignment of the artificial intraocular implant within the pseudophakic eye [7,9,10]. But in case of healthy eyes, when PIII image is missing in the captured visualizations (see Introduction section for detailed explanation), the only quantitative data that can be available directly from Purkinje imaging is limited to the relative distance between PI and PIV. Consequently, no exact estimates of lens tilt and decentration can be obtained, but rather an infinite set of tilt-decentration pairs matching a particular Purkinje performance, as given by Eq. (2). This means that from a particular Purkinje performance (a particular PIV-PI configuration), one cannot estimate a single solution of crystalline lens tilt and decentration values. This claim was previously concluded by He et al. [3]. On the basis of Purkinje image simulations they demonstrated a linear dependencies between the PIV-PI distance and the lens tilt and lens decentration. In the present study such a dependency was confirmed and extended to a planar three dimensional function. This way we demonstrated that the function $\Delta x_{PIV-PI}=f(tilt,dec)$ is explicitly planar, and – as a consequence – many different combinations of crystalline lens spatial conformation may provide the same optical effect in terms of Purkinje performance (compare: Visualization 1 and Visualization 2).

Under that reasoning, it is very unlikely that either pure lens decentration or pure lens inclination are responsible for crystalline lens inertial motion pattern, since these are only two out of a number of possible combinations that may result in the exactly the same Purkinje performance. And pure tilt and pure decentration are just two of possible solutions, the most extreme ones. As it was claimed by He et al. [3] and Tabernero et al. [13], it is more probable that both tilt and decentration contribute to the overall lens wobbling, leading to more complex description of the motion.

In order to investigate the complexity of crystalline lens wobbling motion, we performed combined mechanical and optical simulations, and the data and computation workflow followed the diagram presented in Fig. 5. In both types of simulations we implemented a simple opto-mechanical model of the eye, taking into account the physiological values of biomechanical and optical parameters. Two extreme values of the Young's moduli of zonular fibres were taken into account. The results for them may be significant, as the moduli can vary with age [26].

By using mixed mechanical and optical computation tools, it was possible to estimate the time evolutions associated to both lens tilt and decentration. It has been demonstrated that the lens wobbling is a result of complex entanglement of both the lens tilt and decentration.

The results are presented for two-dimensional mechanical model of the eye. Although this model simplifies the problem to lens movements in one direction and does not take into account complex phenomena, such as the flow of aqueous humor through the spaces between ligaments, it requires much less computational power than 3D model. However, these simplifications may affect the obtained results, such as the amplitude magnitude or frequency of lens oscillations.

Since one cannot observe the lens wobbling directly by any available experimental techniques, but rather its optical effect by means of Purkinje imaging, our computation approach enabled to estimate the optical effect introduced by superposition of tilt and decentration in terms of effective Purkinje PIV-PI pattern. At a glance, a simple relation can be observed: the stiffer the zonules, the slower the wobbling amplitude vanishes, and the larger is the frequency of oscillations. A closer look at the patterns reveals that the tilt and decentration seem to be slightly out of phase (see Table 4).

Visualization 3 clearly demonstrates how complex is the nature of the lens wobbling motion, in terms of relative displacement of the mesh within the lens from its balanced (final) position. One can notice that the brightest spot associated with the least mesh displacement (likely being the effective center of lens rotation at a particular moment) is rapidly moving along the horizontal axis of the eye, from left to right and from right to left.

The simulated effective Purkinje performance of the crystalline lens wobbling is smooth and regular, and noticeably resembles the PIV-PI pattern obtained in *in vivo* measurements in healthy eyes [1] (see the lower panel of Fig. 8), with comparable amplitude decay (especially for E = 0.279 MPa). However the frequency of wobbling oscillations still appears to be significantly higher. It is very likely that some other mechanical parameters – out of the ones listed in Table 1 - have more significant impact on the wobbling frequency, or even non-homogeneity of the mechanical properties of the several structural needs to be taken into account. But it needs to be emphasized that development of such an opto-mechanical model was not in the scope of the present study. We decided to adapt the optical model [14] based on ocular biometry and use it together with the mechanical data found in the literature [15–23]. It was meant to be used simply as an utensil for presentation of the computation methodology. Therefore at this stage of the study it was not meant to be optimized (in terms of geometry and mechanical properties of the structures) to precisely meet the Purkinje pattern parameters of a living human eye [1]. However the workflow presented in this research may be the basis for development of a versatile opto-mechanical eye model that would meet the lens wobbling pattern criteria for optimization.

Up to now the wobbling phenomenon has been considered as a curiosity or an interesting fact, rather than a source of quantitative information on the condition of the optical system of the eye. Although the influence of ciliary muscle tension [13] and accommodation on the wobbling pattern has been proved [3], it is still treated as “artifact”.

We believe that further investigations on complexity of wobbling motion may be targeted to description of the influence of the mechanical properties of ocular structures on effective lens wobbling motion pattern i.e. by employing machine learning and optimization methods, that would make it possible to quantify these effects. The potential of this approach is still to be discovered, however it might find the wobbling phenomenon to be used as a kind of an *in situ* biomarker of the conditions of the ocular system. This might be of particular importance of investigations i.e. aimed to design, optimize the haptics of the intraocular lenses with regard to its stability, and enable further development of the ocular implantology.

## 5. Conclusions

To the best of our knowledge, no research on possible co-existence of these basic motions in the complex wobbling process has been discussed in detail. The approach to the phenomenon so far has been somewhat simplified. Tabernero et al., in their earlier study on the wobbling phenomenon [1], have simulated the retinal image during wobbling by implementing only the lens decentration, which is rather unlikely in the real conditions. However, in more recent paper, they claimed that wobbling might be the combined effect of lens tilt and decentration [13], as previously suggested by He et al. [3]. The present numerical investigation confirmed and significantly extended these studies, proving the intuitive claim that both the tilt and decentration contribute in the wobbling motion and their mutual superposition plays an important role in the effective Purkinje performance which is the only measurable effect of the crystalline lens wobbling.

This work also presents a computation methodology that employs both the mechanical and optical simulation tools. This combination seems to be of increasing interest of the research in the field of physiological optics, due to its ability to model the influence of structural changes induced by dynamic mechanical processes in biological tissues on the optical performance [30,31]. Our investigations aimed to provide a reliable Purkinje performance data, that takes into account the complex nature of wobbling motion. We believe that this data and computation workflow will provide a powerful, practical tool for further research on crystalline lens wobbling.

## Data Availability

Data underlying the results presented in this paper are not publicly available at this time but may be obtained from the authors upon reasonable request.

## References

[1] Tabernero J. Artal P., “Lens oscillations in the human eye: implications for post-saccadic suppression of vision,” PLoS One 9(4), e95764 (2014). 10.1371/journal.pone.0095764 24755771 PMC3995773

[2] Deubel H. Bridgeman B., “Fourth Purkinje image signals reveal eye-lens deviations and retinal image distortions during saccades,” Vision Res. 35(4), 529–538 (1995). 10.1016/0042-6989(94)00146-D 7900293

[3] He L. Donnelly W. J. Stevenson S. B. Glasser A., “Saccadic lens instability increases with accommodative stimulus in presbyopes,” J Vis 10(4), 1–16 (2010). 10.1167/10.4.14 PMC2913422 20465334

[4] Navarro R. Méndez-Morales J. A. Santamaría J., “Optical quality of the eye lens surfaces from roughness and diffusion measurements,” J. Opt. Soc. Am. A 3(2), 228–234 (1986). 10.1364/JOSAA.3.000228 3950796

[5] Millodot M., *Dictionary of Optometry and Visual Science E-Book* (Elsevier Health Sciences, 2014).

[6] Santos P. Martínez-Roda J. A. Ondategui J. C. Díaz-Doutón F. Cazal J. A. O. Vilaseca M., “System based on the contrast of Purkinje images to measure corneal and lens scattering,” Biomed. Opt. Express 9(10), 4907 (2018). 10.1364/BOE.9.004907 30319911 PMC6179394

[7] de Castro A. Rosales P. Marcos S., “Tilt and decentration of intraocular lenses in vivo from Purkinje and Scheimpflug imaging,” J. Cataract Refract. Surg. 33(3), 418–429 (2007). 10.1016/j.jcrs.2006.10.054 17321392

[8] Rosales P. de Castro A. Jiménez-Alfaro I. Marcos S., “Intraocular lens alignment from Purkinje and Scheimpflug imaging,” Clin. Exp. Optom. 93(6), 400–408 (2010). 10.1111/j.1444-0938.2010.00514.x 20738324

[9] Tabernero J. Benito A. Nourrit V. Artal P., “Instrument for measuring the misalignments of ocular surfaces,” Opt. Express 14(22), 10945 (2006). 10.1364/OE.14.010945 19529508

[10] Jóźwik A. Siedlecki D. Zając M., “Analysis of Purkinje images as an effective method for estimation of intraocular lens implant location in the eyeball,” Optik (Munich, Ger.) 125(20), 6021–6025 (2014). 10.1016/j.ijleo.2014.06.130

[11] Maedel S. Hirnschall N. Bayer N. Markovic S. Tabernero J. Artal P. Schaeffel F. Findl O., “Comparison of intraocular lens decentration and tilt measurements using 2 Purkinje meter systems,” J. Cataract Refract. Surg. 43(5), 648–655 (2017). 10.1016/j.jcrs.2017.01.022 28602327

[12] Eto T. Teikari P. Najjar R. P. Nishimura Y. Motomura Y. Kuze M. Higuchi S., “A Purkinje image-based system for an assessment of the density and transmittance spectra of the human crystalline lens in vivo,” Sci. Rep. 10(1), 16445 (2020). 10.1038/s41598-020-73541-y 33020575 PMC7536217

[13] Tabernero J. Chirre E. Hervella L. et al., “The accommodative ciliary muscle function is preserved in older humans,” Sci. Rep. 6(1), 25551 (2016). 10.1038/srep25551 27151778 PMC4858807

[14] Zapata-Díaz J. F. Radhakrishnan H. Charman W. N. López-Gil N., “Accommodation and age-dependent eye model based on in vivo measurements,” J. Optom. 12(1), 3–13 (2019). 10.1016/j.optom.2018.01.003 29573985 PMC6318498

[15] Ludwig K., “Zonular apparatus, anatomy, biomechanics and coupling to the lens,” In: Guthoff R. Ludwig K., eds. *Current Aspects of Human Accommodation* (Kaden, 2001).

[16] Lanchares E. Navarro R. Calvo B., “Hyperelastic modelling of the crystalline lens: Accommodation and presbyopia,” J. Optom. 5(3), 110–120 (2012). 10.1016/j.optom.2012.05.006

[17] Dai P. Zhao Y. Sheng H. Li L. Wu J. Han H., “Simulating the effects of elevated intraocular pressure on ocular structures using a global finite element model of the human eye,” J. Mech. Med. Biol. 17(02), 1750038 (2017). 10.1142/S0219519417500385

[18] Ayyalasomayajula A. Park R. I. Simon B. R. Vande Geest J. P., “A porohyperelastic finite element model of the eye: the influence of stiffness and permeability on intraocular pressure and optic nerve head biomechanics,” Comput. Methods Biomech. Biomed. Engin. 19(6), 591–602 (2016). 10.1080/10255842.2015.1052417 26195024 PMC4721930

[19] Issarti I. Koppen C. Rozema J. J., “Influence of the eye globe design on biomechanical analysis,” Comput. Biol. Med. 135, 104612 (2021). 10.1016/j.compbiomed.2021.104612 34261005

[20] Weeber H. A. van der Heijde G. L., “On the relationship between lens stiffness and accommodative amplitude,” Comput. Biol. Med. 85(5), 602–607 (2007). 10.1016/j.exer.2007.07.012 17720158

[21] Burd H. J. Judge S. J. Cross J. A., “Numerical modelling of the accommodating lens,” Vision Res. 42(18), 2235–2251 (2002). 10.1016/S0042-6989(02)00094-9 12207982

[22] Bocskai Z. Bojtár I., “Biomechanical modelling of the accommodation problem of human eye,” Per. Pol. Civil Eng. 57(1), 3–9 (2013). 10.3311/PPci.2136

[23] Liu Z. Wang B. Xu X. Ju Y. Xie J. Bao C., “Finite element modeling and simulating of accommodating human crystalline lens,” in: Engineering in Medicine and Biology Society, 2005. IEEE-EMBS 2005. 27th Annual International Conference of the*, pp.* 11–14. 10.1109/IEMBS.2005.1616329 17282098

[24] Scott J. A., “A finite element model of heat transport in the human eye,” Phys. Med. Biol. 33(2), 227–242 (1988). 10.1088/0031-9155/33/2/003 3362966

[25] Ooi E. H. Ng E. Y., “Simulation of aqueous humor hydrodynamics in human eye heat transfer,” Comput. Biol. Med. 38(2), 252–262 (2008). 10.1016/j.compbiomed.2007.10.007 18022147

[26] Michael R. Mikielewicz M. Gordillo C. Montenegro G. A. Pinilla Cortés L. Barraquer R. I., “Elastic properties of human lens zonules as a function of age in presbyopes,” Invest Ophthalmol Vis Sci. 53(10), 6109–6114 (2012). 10.1167/iovs.11-8702 22850416

[27] van Alphen G. Graebel W. P., “Elasticity of tissues involved in accommodation,” Vision Res. 31(7-8), 1417–1438 (1991). 10.1016/0042-6989(91)90061-9 1891828

[28] OpticStudio, version 22.1.1, Zemax LLC, Kirkland, WA.

[29] Collewijn H. Erkelens C. J. Steinman R. M., “Binocular co-ordination of human horizontal saccadic eye movements,” J. Physiol. 404(1), 157–182 (1988). 10.1113/jphysiol.1988.sp017284 3253429 PMC1190820

[30] Wang K. Venetsanos D. T. Hoshino M. Uesugi K. Yagi N. Pierscionek B. K., “A modeling approach for investigating opto-mechanical relationships in the human eye lens,” IEEE Trans. Biomed. Eng. 67(4), 999–1006 (2020). 10.1109/TBME.2019.2927390 31395531

[31] Cabeza-Gil I. Grasa J. Calvo B., “A validated finite element model to reproduce Helmholtz’s theory of accommodation: a powerful tool to investigate presbyopia,” Ophthalmic Physiol Opt. 41(6), 1241–1253 (2021). 10.1111/opo.12876 34463367

